# Supplementary material for: Prevalence and Persistence of Ceftiofur-Resistant Escherichia coli in A Chicken Layer Breeding Program
Source: Animals (Basel). 2022 Dec 26;13(1):90. doi: 10.3390/ani13010090 (PMC9817529; doi:10.3390/ani13010090)
Supplement: Supplementary file 1 [file animals-13-00090-s001.zip › animals-2044390-supplementary/animals-2044390 Supplementary file/Supplementary Figure S1.pdf]

S1

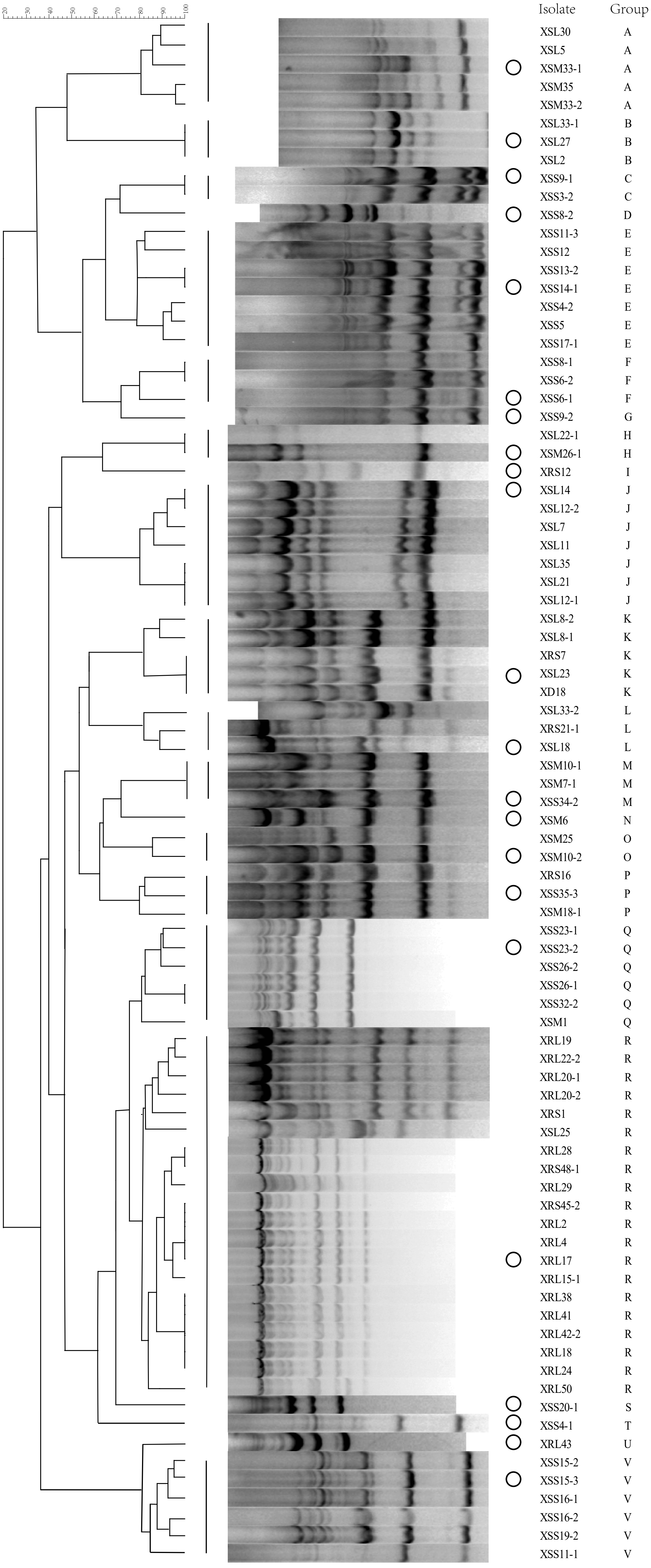
20
30
40
50
60
70
80
90
100
Isolate
Group
XSL30 A
XSL5 A
XSM33-1 A
XSM35 A
XSM33-2 A
XSL33-1 B
XSL27 B
XSL2 B
XSS9-1 C
XSS3-2 C
XSS8-2 D
XSS11-3 E
XSS12 E
XSS13-2 E
XSS14-1 E
XSS4-2 E
XSS5 E
XSS17-1 E
XSS8-1 F
XSS6-2 F
XSS6-1 F
XSS9-2 G
XSL22-1 H
XSM26-1 H
XRS12 I
XSL14 J
XSL12-2 J
XSL7 J
XSL11 J
XSL35 J
XSL21 J
XSL12-1 J
XSL8-2 K
XSL8-1 K
XRS7 K
XSL23 K
XD18 K
XSL33-2 L
XRS21-1 L
XSL18 L
XSM10-1 M
XSM7-1 M
XSS34-2 M
XSM6 N
XSM25 O
XSM10-2 O
XRS16 P
XSS35-3 P
XSM18-1 P
XSS23-1 Q
XSS23-2 Q
XSS26-2 Q
XSS26-1 Q
XSS32-2 Q
XSM1 Q
XRL19 R
XRL22-2 R
XRL20-1 R
XRL20-2 R
XRS1 R
XSL25 R
XRL28 R
XRS48-1 R
XRL29 R
XRS45-2 R
XRL2 R
XRL4 R
XRL17 R
XRL15-1 R
XRL38 R
XRL41 R
XRL42-2 R
XRL18 R
XRL24 R
XRL50 R
XSS20-1 S
XSS4-1 T
XRL43 U
XSS15-2 V
XSS15-3 V
XSS16-1 V
XSS16-2 V
XSS19-2 V
XSS11-1 V
